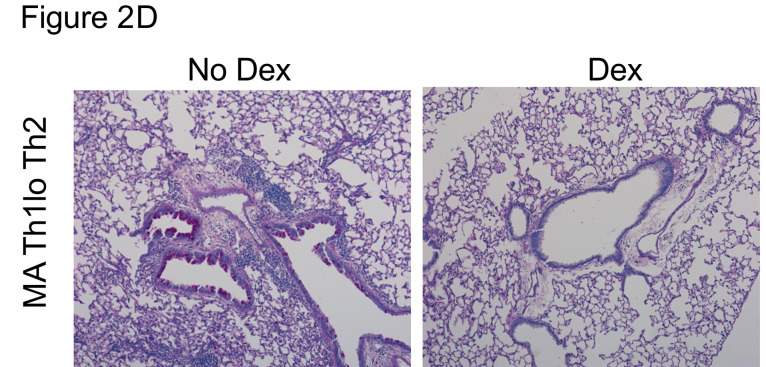# Corrigendum to High IFN-γ and low SLPI mark severe asthma in mice and humans

**DOI:** 10.1172/JCI195762

**Published:** 2025-07-01

**Authors:** Mahesh Raundhal, Christina Morse, Anupriya Khare, Timothy B. Oriss, Jadranka Milosevic, John Trudeau, Rachael Huff, Joseph Pilewski, Fernando Holguin, Jay Kolls, Sally Wenzel, Prabir Ray, Anuradha Ray

Original citation: *J Clin Invest*. 2015;125(8):3037–3050. https://doi.org/10.1172/JCI80911

Citation for this corrigendum: *J Clin Invest*. 2025;135(13):e195762. https://doi.org/10.1172/JCI195762

The authors recently became aware that the MA Th1^lo^Th2 No Dex image in Figure 2D is the same as the WT SA + Dex image in Figure 6C. The authors were unable to retrieve the original source data but have provided recent data from the same models with and without Dex inhibition to replace images in these panels. The correct images are shown below.

The authors regret the error.

## Figures and Tables

**Figure 6C:**
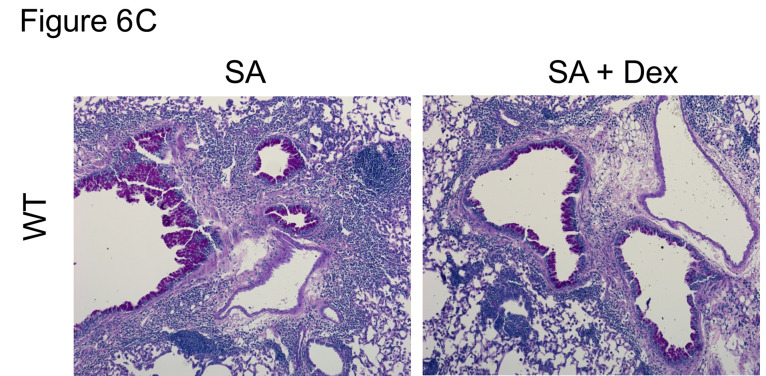


**Figure 2D:**